# Average log change rate of pretreatment squamous cell carcinoma antigen after concurrent chemoradiotherapy in stage IIIC1 cervical squamous cell carcinoma

**DOI:** 10.1038/s41598-024-59412-w

**Published:** 2024-04-15

**Authors:** Oyeon Cho, Mison Chun, Suk-Joon Chang

**Affiliations:** 1 Gynecologic Cancer Center, Department of Radiation Oncology, Ajou University of School of Medicine, 164, World cup-ro, Yeongtong-gu, Suwon, 16499 Korea; 2 Gynecologic Cancer Center, Department of Obstetrics and Gynecology, Ajou University of School of Medicine, Suwon, 16499 Korea

**Keywords:** Cancer, Predictive markers

## Abstract

We aimed to determine whether pretreatment squamous cell carcinoma antigen (SCC-Ag) levels and the average logarithmic change in SCC-Ag levels ($\frac{{\Delta \log \left( {{\text{SCC-Ag}}} \right)}}{{\Delta {\text{time}}}}$) after concurrent chemoradiotherapy (CCRT) could predict treatment outcomes in patients with stage IIIC1 cervical squamous cell carcinoma (SCC). We analyzed 168 patients with stage IIIC1 cervical SCC who underwent primary CCRT and collected data on age, local extension, treatment details, hematological parameters, and tumor markers such as SCC-Ag and carcinoembryonic antigen 21-1 (Cyfra). Predictive performances of pretreatment SCC-Ag levels and $\frac{{\Delta \log \left( {{\text{SCC-Ag}}} \right)}}{{\Delta {\text{time}}}}$ were assessed using receiver operating characteristic curves. Survival analysis was performed using the Cox regression model and Kaplan–Meier plots. The combination of pretreatment SCC-Ag levels and $\frac{{\Delta \log \left( {{\text{SCC-Ag}}} \right)}}{{\Delta {\text{time}}}}$ showed higher area under the curve values than pretreatment SCC-Ag levels alone (area under the curve; 95% confidence interval [CI] 0.708 [0.581–0.836] vs. 0.666 [0.528–0.804], respectively). Pretreatment SCC-Ag (≥ 5 ng/ml and Cyfra levels (≥ 3.15 ng/ml) and $\frac{{\Delta \log \left( {{\text{SCC-Ag}}} \right)}}{{\Delta {\text{time}}}}$ (≥ − 1.575) were significant predictors of disease-specific survival. The 5-year disease-specific survival rates significantly differed among the low-, intermediate-, and high-risk groups. Risk stratification using both pretreatment SCC-Ag levels and $\frac{{\Delta \log \left( {{\text{SCC-Ag}}} \right)}}{{\Delta {\text{time}}}}$ may predict treatment outcomes of patients with stage IIIC1 SCC.

## Introduction

The International Federation of Gynecology and Obstetrics (FIGO) cervical cancer staging system, revised in 2018, includes radiologically or pathologically detected lymph node (LN) metastases in the pelvic (IIIC1) or para-aortic region (IIIC2) as a criterion^1^. Two studies that compared this staging system with the 2009 FIGO staging system emphasized the varied treatment outcomes of patients with stage IIIC cancer when all patients with LN metastases were grouped together^2,3^. Notably, both reports demonstrated that patients with stage IIIC1 cancer had a higher 5-year survival rate than patients with stage IIIA or IIIB cancer (76.3 vs. 22.8 or 38.6% and 60.8 vs. 40.7 or 41.4%, respectively). However, one study indicated that patients with stage IIIC1 cancer had a higher 5-year survival rate than patients with stage IIB cancer (76.3 vs. 66.3%, respectively), whereas another study reported a lower 5-year survival rate in patients with stage IIIC1 cancer than in patients with stage IIB cancer (60.8 vs. 63.9%, respectively)^2,3^. Furthermore, another study reported no significant difference in the 5-year survival rates between patients with stage IIIC1 and those with stages IIIA and IIIB cancer (71.0 vs. 67.6%, *P* = 0.498)^4^. In another study, pelvic LN metastasis, which was detected through imaging studies (stage IIIC1-r), could not help in differentiating the 5-year survival rates between patients with stage I or II and those with stage IIIC1-r cancer (98.8 or 97.7 vs. 96.9%, respectively; *P* = 0.21)^5^. The variability in the prognosis of stage IIIC1 cancer may be attributed to insufficient information regarding the disease extent, despite the prognostic impact of pelvic LN metastasis^6^. Therefore, it is necessary to utilize existing biomarkers to predict the treatment outcomes of stage IIIC1 cancer and establish an appropriate treatment strategy.

The squamous cell carcinoma antigen (SCC-Ag) is a widely used biomarker for predicting treatment outcomes and monitoring tumor recurrence in patients with cervical cancer undergoing concurrent chemoradiotherapy (CCRT)^7^. Specifically, a meta-analysis revealed that an increase in pre- and post-treatment SCC-Ag levels (pre-SCC-Ag and post-SCC-Ag, respectively) is associated with tumor recurrence or death^8^. Additionally, the rate of reduction in SCC-Ag levels during CCRT has been linked to treatment outcomes^9,10^. These findings suggest that pre-SCC-Ag levels and the post-treatment reduction rate can reflect the disease extent and CCRT response, respectively. Therefore, we assumed that an exponential decrease in SCC-Ag levels may provide an accurate indication of the CCRT response. The reason for this assumption was that SCC-Ag, which is produced through the neoplastic transformation of squamous epithelium, shows an exponential distribution pattern similar to that of the cell survival curves showing cell survival rates at different radiation doses, as determined through time-lapse imaging^11,12^.

Accordingly, we aimed to investigate whether pre-SCC-Ag levels and the average log change rate after treatment could predict the treatment outcomes in stage IIIC1 cervical SCC patients treated with CCRT.

## Methods

### Patient selection

This study received approval from the Institutional Review Board of Ajou University Hospital (approval number: DB-2023-336), which waived the requirement for informed consent due to the retrospective nature of the study. All procedures were conducted in accordance with the Declaration of Helsinki. Among 207 patients who were diagnosed with stage IIIC1-r cervical SCC (converted to the 2018 FIGO stage for patients who were diagnosed before 2018) and treated with primary weekly cisplatin-based CCRT at our institution from April 2001 to December 2021, 168 patients were included in the analysis. The following patients were excluded: (1) patients who received < 50 Gy total dose (TD); (2) those with a follow-up period < 12 months after the end of treatment; (3) those whose tumor marker (TM) levels, including SCC-Ag and Cyfra levels, were not measured before, during, and after treatment; and (4) those whose disease had progressed before post-treatment TM measurement.

### Patients

All patients were confirmed to have invasive SCC through cervical biopsy. Local progression, LN metastasis, and distant metastasis (DM) were evaluated using physical examination, magnetic resonance imaging (MRI), computed tomography (CT), and positron emission tomography-CT (PET-CT). Cystoscopy or sigmoidoscopy was performed when bladder or rectal invasion was suspected. Pelvic external beam radiation therapy (EBRT) was delivered at a dose of 45 Gy in 25 fractions; moreover, the enlarged lymph nodes were boosted to 55–60 Gy. Overall, 166 patients underwent high-dose intracavitary brachytherapy, with doses of 8–30 Gy being delivered in 2–7 fractions at point A (using Iridium-192; MicroSelectron, Nucletron, Veenendaal, Netherlands or GammaMedplus iX, Varian, Palo Alto, CA, USA). Two patients were treated with EBRT alone. The point defined by the International Commission on Radiation Units and Measurements (Report 38) was modified to 1.0–1.8 cm based on the size of the uterus. Cisplatin (30–70 mg/m^2^) was administered weekly for 5–6 cycles during radiation therapy (RT). All patients did not receive any additional treatment before or after CCRT. Follow-up was conducted every 1–3 months after treatment; moreover, local progression, regional LN metastasis, and LN metastasis were evaluated using a Pap smear test, analysis of levels of TM such as SCC-Ag and Cyfra, CT, and MRI. Patients who showed disease progression received chemotherapy (CTx), CCRT, or conservative care. The CTx regimens included combinations of 5-fluorouracil; topotecan; paclitaxel and cisplatin; or paclitaxel, cisplatin, and bevacizumab.

### Variables

Data on the following variables were collected from all patients: age at diagnosis, local extension (LE), TD, treatment time (TT), pretreatment neutrophil-to-lymphocyte ratio (NLR), pretreatment hemoglobin (Hb) levels, pretreatment platelet (PLT) levels, pre-SCC-Ag, pretreatment Cyfra levels (pre-Cyfra), SCC-Ag during CCRT (mid-SCC-Ag), Cyfra during CCRT (mid-Cyfra), post-SCC-Ag, post-treatment Cyfra (post-Cyfra), and the date when TM levels was measured relative to the date of treatment initiation. LE was divided into the following three groups: confined to the cervix, invading the parametrium, and invading the pelvic wall or lower vagina (PW/LV). TD was the sum of EBRT and brachytherapy doses directed toward the central lesion (equivalent dose in 2 Gy fractions, α/β = 10).

### Average rate of log change

Assuming that TM levels decrease exponentially with radiation in a manner similar to the pattern shown by cancer cell survival curves (Eq. 1), the rate of log change per time can be calculated using the pre-treatment TM level (TM_0_) and the TM level (TM_1_) at a specific time point (t_1_) after the treatment initiation (t_0_). Mathematically, this equation represents the slope (− α) of the exponential function (Eq. 2).

1 $$ {\text{y}} = {\text{e}}^{{ - \upalpha {\text{x}}}} \to \log y = - \alpha x. $$

2 $$ \log \left( {{\text{TM}}_{1} } \right) - {\text{log}}\left( {{\text{TM}}_{0} } \right) = - \alpha \left( {t_{1} - t_{0} } \right) \to \frac{{\Delta \log \left( {{\text{TM}}} \right)}}{{\Delta {\text{time}}}} = - \alpha . $$

We defined the average rate of log change, multiplied by 100, between the pretreatment TM level and the TM level during CCRT as $\frac{{\Delta \log \left( {{\text{TM}}} \right)}}{{\Delta {\text{time}}}}$(mid) and the average rate of log change, multiplied by 100, between the pretreatment and posttreatment TM levels as $\frac{{\Delta \log \left( {{\text{TM}}} \right)}}{{\Delta {\text{time}}}}{ }\left( {{\text{post}}} \right)$. If TM was measured as 0, it was replaced by 0.01 for log calculation. We applied these definitions to SCC-Ag and Cyfra, resulting in calculations such as $\frac{{\Delta \log \left( {{\text{SCC-Ag}}} \right)}}{{\Delta {\text{time}}}}$(mid) or $\frac{{\Delta \log \left( {{\text{Cyfra}}} \right)}}{{\Delta {\text{time}}}}$(post). All specific measurement times for TMs were described in the dataset posted online.

### Receiver operating characteristic curves

We evaluated the predictive performance of various parameters for treatment outcomes by combining pretreatment TM levels with the rate of change in TM levels. We analyzed the area under the curve (AUC) values and 95% confidence intervals (CIs) of the receiver operating characteristic (ROC) curves for the following seven groups:

1. Pre-SCC-Ag versus pre-SCC-Ag + $\frac{{\Delta \log \left( {{\text{SCC-Ag}}} \right)}}{{\Delta {\text{time}}}}$(mid) or pre-SCC-Ag + $\frac{{\Delta \log \left( {{\text{SCC-Ag}}} \right)}}{{\Delta {\text{time}}}}$(post)
2. Pre-Cyfra versus pre-Cyfra + $\frac{{\Delta \log \left( {{\text{Cyfra}}} \right)}}{{\Delta {\text{time}}}}$(mid) or pre-Cyfra + $\frac{{\Delta \log \left( {{\text{Cyfra}}} \right)}}{{\Delta {\text{time}}}}$(post)
3. Pre-SCC-Ag versus pre-Cyfra or pre SCC-Ag + pre-Cyfra
4. Pre-SCC-Ag versus pre-SCC-Ag + $\frac{{\Delta {\text{SCC-Ag}}}}{{{\text{pre SCC}} - {\text{Ag}}}}$(mid) or pre-SCC-Ag + $\frac{{\Delta {\text{SCC-Ag}}}}{{{\text{pre SCC}} - {\text{Ag}}}}\left( {{\text{post}}} \right)$
5. Pre-Cyfra versus pre-Cyfra + $\frac{{\Delta {\text{Cyfra}}}}{{\text{pre Cyfra}}}$(mid) or pre-Cyfra + $\frac{{\Delta {\text{Cyfra}}}}{{\text{pre Cyfra}}}\left( {{\text{post}}} \right)$
6. Pre-SCC-Ag versus pre-SCC-Ag + $\frac{{\Delta {\text{SCC-Ag}}}}{{\Delta {\text{time}}}}$(mid) or pre-SCC-Ag + $\frac{{\Delta {\text{SCC-Ag}}}}{{\Delta {\text{time}}}}\left( {{\text{post}}} \right)$
7. Pre-Cyfra versus pre-Cyfra + $\frac{{\Delta {\text{Cyfra}}}}{{\Delta {\text{time}}}}$(mid) or pre-Cyfra + $\frac{{\Delta {\text{Cyfra}}}}{{\Delta {\text{time}}}}\left( {{\text{post}}} \right)$

The AUC and 95% CI values were calculated using the DeLong test. Multiple logistic regression was used for the ROC curve analysis, including two variables. The Youden index was calculated as sensitivity + specificity − 1.

### Survival analysis

We conducted a comparative analysis using the χ^2^ test to examine differences in disease-specific death (DSD), progression, age at diagnosis, LE, TD, TT, NLR, Hb levels, mid-SCC-Ag, post-SCC-Ag, $\frac{{\Delta \log \left( {{\text{SCC-Ag}}} \right)}}{{\Delta {\text{time}}}}$(mid), pre-Cyfra, mid-Cyfra, post-Cyfra, $\frac{{\Delta \log \left( {{\text{Cyfra}}} \right)}}{{\Delta {\text{time}}}}$(mid), and $\frac{{\Delta \log \left( {{\text{Cyfra}}} \right)}}{{\Delta {\text{time}}}}$(post) among three groups of patients categorized into low-, intermediate-, and high-risk groups based on the median values of pre-SCC-Ag and $\frac{{\Delta \log \left( {{\text{SCC-Ag}}} \right)}}{{\Delta {\text{time}}}}$(post) using the χ^2^ test. The primary endpoints of our analysis were disease-specific survival (DSS) and progression-free survival (PFS). The follow-up period was measured from the end of treatment to the last visit or event date. Univariate and multivariate analyses were performed using backward elimination, including all candidate variables, except for a noise variable. We used Kaplan–Meier curves and log-rank tests to compare the 5-year DSS (5DSS) and 5-year PFS (5PFS) rates among the three patient groups.

All data analyses and visualizations were performed using R version 4.2.3 (https://www.r-project.org).

## Results

### Tumor marker changes over time

**Figure 1:**
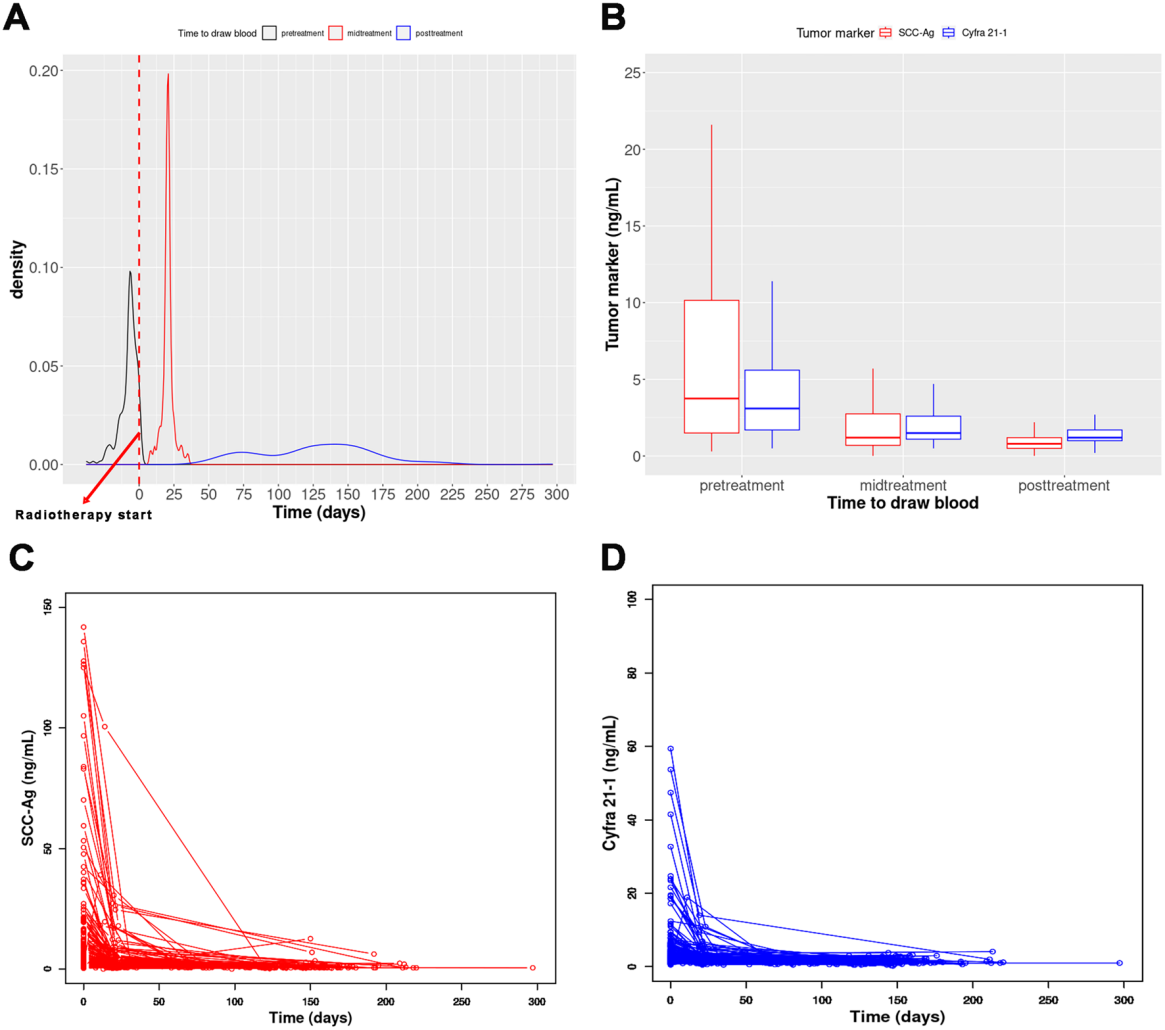
(**A**) Density plot showing distribution of measured time. (**B**) Box plots displaying distribution of SCC-Ag and Cyfra levels before treatment (pretreatment), during treatment (mid-treatment), and after treatment (post-treatment). (**C**) and (**D**) Changes in SCC-Ag and Cyfra levels over time, respectively. The measured time of pretreatment tumor marker analysis was considered zero in these plots. *SCC-Ag* squamous cell carcinoma antigen, *Cyfra* carcinoembryonic antigen 21-1.

Figure 1 shows the quantitative changes in TM levels over time. The slope between the pretreatment TM level and TM level during CCRT (mid-treatment) was lower than that between the mid-treatment and post-treatment TM levels. The measurement timing of post-treatment TM levels varied from 47 to 297 days after CCRT initiation. TM levels decreased exponentially with time after treatment.

### ROC curves for tumor markers and their change rate

**Figure 2:**
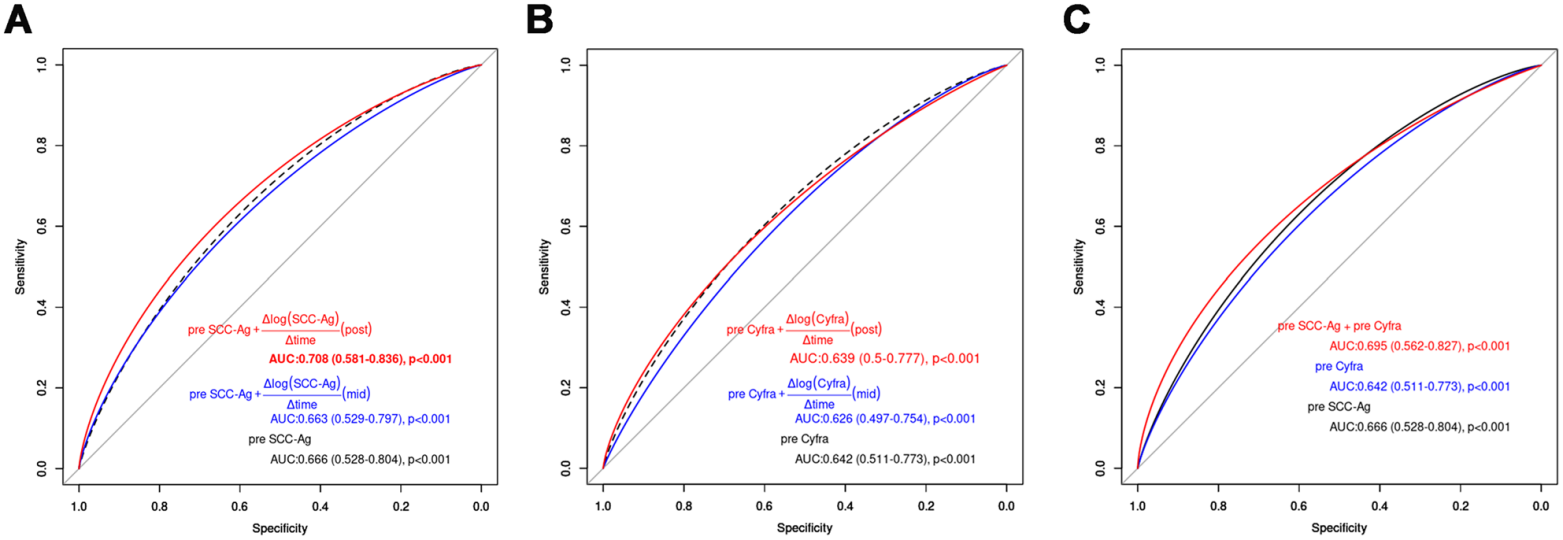
Receiver operating characteristic curves and their corresponding AUCs with 95% confidence intervals. (**A**) Pre-SCC-Ag and pre-SCC-Ag + average log change rates during and after treatment. (**B**) Pre-Cyfra and pre-Cyfra + average log change rates during and after treatment. (**C**) Pre-SCC-Ag, pre-Cyfra, and pre-SCC-Ag + pre-Cyfra. *AUC* area under the curve, *SCC-Ag* squamous cell carcinoma antigen, *Cyfra* carcinoembryonic antigen 21-1, *pre-SCC-Ag* pretreatment SCC-Ag, *pre-Cyfra* pretreatment Cyfra.

Analysis of all possible combinations in the seven groups described in the Methods section revealed that no pairs showed statistically significant differences in the AUC of ROC curves (Table S1). However, the combination of pre-SCC-Ag and the average rate of log change in pre-SCC-Ag, as well as the combination of pre-SCC-Ag and pre-Cyfra, exhibited higher AUC and Youden index values than pre-SCC-Ag alone. Specifically, the AUC and Youden index values for the combination of pre-SCC-Ag and the average rate of log change in pre-SCC-Ag were 0.708 and 0.466, respectively, while those for pre-SCC-Ag alone were 0.666 and 0.385, respectively. This information is presented in Fig. 2, Fig. S1, and Table S1.

### Patient characteristics

Table 1 presents the patient characteristics and compares the differences in variables among the three patient groups categorized according to risk. The high-risk group had significantly higher DSD rates than the low- and intermediate-risk groups, which could be mainly attributed to the higher occurrence of DM in the high-risk group than in the other groups. The variables mid-SCC-Ag, post-SCC-Ag, and $\frac{{{\Delta }\log \left( {{\text{Cyfra}}} \right)}}{{\Delta {\text{time}}}}$(post) were significantly associated with this risk stratification.

**Table 1:** Patients’ characteristics based on squamous cells carcinoma antigen and its average log change rate.

Factors	Median (range) or N (%)	Low	Intermediate	High	
Pre SCC-Ag	5 (0.3–141.8)	< 5	≥ 5 (< 5)	≥ 5	
$\frac{{\Delta \log \left( {{\text{SCC-Ag}}} \right)}}{{\Delta {\text{time}}}}$(post)	− 1.575 (− 7.261 to 1.029)	< − 1.575	< − 1.575 (≥ − 1.575)	≥ − 1.575	

	(N = 168)	(N = 11)	(N = 145)	(N = 12)	*P*
Disease specific death					0
No	148 (88.1%)	11 (100.0%)	131 (90.3%)	6 (50.0%)	
Yes	20 (11.9%)	0 (0%)	14 (9.7%)	6 (50.0%)	
Progression					0.186
No	131 (78.0%)	10 (90.9%)	115 (79.3%)	6 (50.0%)	
LP	11 (6.5%)	1 (9.1%)	9 (6.2%)	1 (8.3%)	
DM	21 (12.5%)	0 (0.0%)	17 (11.7%)	4 (33.3%)	
LP + DM	5 (3.0%)	0 (0.0%)	4 (2.8%)	1 (8.3%)	
Age (years)	53 (26–84)				0.424
< 65	130 (77.4%)	9 (81.8%)	110 (75.9%)	11 (91.7%)	
≥ 65	38 (22.6%)	2 (18.2%)	35 (24.1%)	1 (8.3%)	
Local extension					0.343
Cervix	26 (15.5%)	2 (18.2%)	24 (16.6%)	0 (0.0%)	
Parametrium	122 (72.6%)	7 (63.6%)	106 (73.1%)	9 (75.0%)	
PW or lower vagina	20 (11.9%)	2 (18.2%)	15 (10.3%)	3 (25.0%)	
Total dose (EQD2)	72.25 (57.61–87.06)				0.338
≥ 70	107 (63.7%)	7 (63.6%)	90 (62.1%)	10 (83.3%)	
< 70	61 (36.3%)	4 (36.4%)	55 (37.9%)	2 (16.7%)	
Treatment time (days)	52.5 (39–78)				0.287
< 56	102 (60.7%)	9 (81.8%)	85 (58.6%)	8 (66.7%)	
≥ 56	66 (39.3%)	2 (18.2%)	60 (41.4%)	4 (33.3%)	
Hemoglobin (g/dl)	11.75 (4.9–14.5)				0.476
≥ 11.75	84 (50.0%)	6 (54.5%)	74 (51.0%)	4 (33.3%)	
< 11.75	84 (50.0%)	5 (45.5%)	71 (49.0%)	8 (66.7%)	
NLR	2.35 (0.78–23.5)				0.751
< 2.35	83 (49.4%)	6 (54.5%)	70 (48.3%)	7 (58.3%)	
≥ 2.35	85 (50.6%)	5 (45.5%)	75 (51.7%)	5 (41.7%)	
Platelet (× 10^3^/µl)	269.5 (114–564)				0.644
< 269.5	84 (50.0%)	4 (36.4%)	74 (51.0%)	6 (50.0%)	
≥ 269.5	84 (50.0%)	7 (63.6%)	71 (49.0%)	6 (50.0%)	
Mid-SCC-Ag (ng/ml)	1.25 (0–100.5)				0.007
< 1.25	84 (50.0%)	9 (81.8%)	73 (50.3%)	2 (16.7%)	
≥ 1.25	84 (50.0%)	2 (18.2%)	72 (49.7%)	10 (83.3%)	
Post-SCC-Ag (ng/ml)	0.8 (0–12.6)				0.008
< 0.8	82 (48.8%)	9 (81.8%)	71 (49.0%)	2 (16.7%)	
≥ 0.8	86 (51.2%)	2 (18.2%)	74 (51.0%)	10 (83.3%)	
$\frac{{\Delta \log \left( {{\text{SCC-Ag}}} \right)}}{{\Delta {\text{time}}}}$(mid)	− 5.524 (− 33.7 to 26.56)				0.112
< − 5.524	84 (50.0%)	7 (63.6%)	68 (46.9%)	9 (75.0%)	
≥ − 5.524	84 (50.0%)	4 (36.4%)	77 (53.1%)	3 (25.0%)	
Pre-Cyfra (ng/ml)	3.15 (0.5–59.4)				0.263
< 3.15	84 (50.0%)	3 (27.3%)	74 (51.0%)	7 (58.3%)	
≥ 3.15	84 (50.0%)	8 (72.7%)	71 (49.0%)	5 (41.7%)	
Mid-Cyfra (ng/ml)	1.5 (0.5–18.9)				0.26
< 1.5	76 (45.2%)	7 (63.6%)	62 (42.8%)	7 (58.3%)	
≥ 1.5	92 (54.8%)	4 (36.4%)	83 (57.2%)	5 (41.7%)	
Post-Cyfra (ng/ml)	1.2 (0.2–4.1)				0.437
< 1.2	76 (45.2%)	3 (27.3%)	68 (46.9%)	5 (41.7%)	
≥ 1.2	92 (54.8%)	8 (72.7%)	77 (53.1%)	7 (58.3%)	
$\frac{{\Delta \log \left( {{\text{Cyfra}}} \right)}}{{\Delta {\text{time}}}}$(mid)	− 2.976 (− 14.6 to 23.03)				0.516
< − 2.976	84 (50.0%)	7 (63.6%)	70 (48.3%)	7 (58.3%)	
≥ − 2.976	84 (50.0%)	4 (36.4%)	75 (51.7%)	5 (41.7%)	
$\frac{{\Delta \log \left( {{\text{Cyfra}}} \right)}}{{\Delta {\text{time}}}}$(post)	− 0.697 (− 4.994 to 1.738)				0.007
< − 0.697	84 (50.0%)	9 (81.8%)	73 (50.3%)	2 (16.7%)	
≥ − 0.697	84 (50.0%)	2 (18.2%)	72 (49.7%)	10 (83.3%)	
Second treatment					0.272
Others	10 (27.0%)	0 (0.0%)	8 (26.7%)	2 (33.3%)	
TP	18 (48.6%)	0 (0.0%)	14 (46.7%)	4 (66.7%)	
TPA	9 (24.3%)	1 (100.0%)	8 (26.7%)	0 (0.0%)	

SCC-Ag: squamous cell carcinoma antigen, Cyfra: carcinoembryonic antigen 21-1, pre-SCC-Ag: pretreatment SCC-Ag, mid-SCC-Ag: SCC-Ag during treatment, post-SCC-Ag: posttreatment SCC-Ag, pre-Cyfra: pretreatment Cyfra, mid-Cyfra: Cyfra during treatment, post-Cyfra: posttreatment Cyfra, $\frac{{\Delta \log \left( {{\text{SCC-Ag}}} \right)}}{{\Delta {\text{time}}}}$ (mid): average log change rate × 100 (pre-SCC-Ag ~ mid-SCC-Ag), $\frac{{\Delta \log \left( {{\text{SCC-Ag}}} \right)}}{{\Delta {\text{time}}}}$ (post): average log change rate × 100 (pre-SCC-Ag ~ post-SCC-Ag), $\frac{{\Delta \log \left( {{\text{Cyfra}}} \right)}}{{\Delta {\text{time}}}}$(mid): average log change rate × 100 (pre-Cyfra ~ mid-Cyfra), $\frac{{\Delta \log \left( {{\text{Cyfra}}} \right)}}{{\Delta {\text{time}}}}$ (post): average log change rate × 100 (pre-Cyfra ~ post-Cyfra), LP: local progression, DM: distant metastasis, PW: pelvic wall, EQD2: equivalent dose in 2 Gy factions, NLR: neutrophil to lymphocyte ratio, TP: paclitaxel + cisplatin, TPA: paclitaxel + cisplatin + bevacizumab.

### Survival analysis results

**Figure 3:**
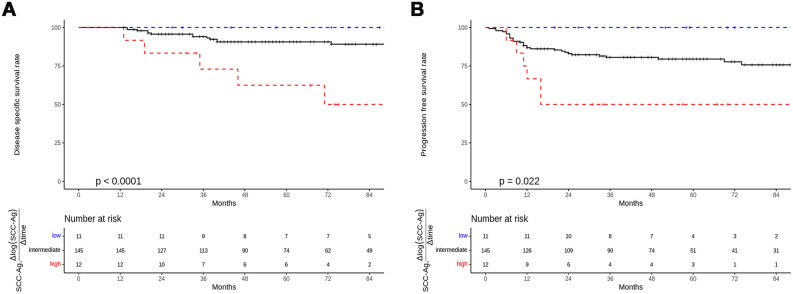
Kaplan–Meier plots of patients categorized into high-, intermediate-, and low-risk groups based on their pre-SCC-Ag levels and the rate of log change in SCC-Ag over time. The plots provide a visual representation of the cumulative survival probability for each group over the course of study. (**A**) Disease-specific survival. (**B**) Progression-free survival. *SCC-Ag* squamous cell carcinoma antigen, *pre-SCC-Ag* pretreatment SCC-Ag.

The median follow-up duration was 62 months (range 13–242 months). The 5DSS and 5PFS rates for all patients in this study were 89.2% and 78.7%, respectively. In the univariate analysis (Table S2), old age and pre-SCC-Ag levels were significant predictors of DSS, whereas PW/LV extension as well as high mid-SCC-Ag and pre-Cyfra levels showed significant hazard ratios for PFS. In the multivariate analysis (Table 2), high pre-SCC-Ag and pre-Cyfra levels and high $\frac{{\Delta \log \left( {{\text{SCC-Ag}}} \right)}}{{\Delta {\text{time}}}}$(post) were significant predictors of DSS, whereas high pre-SCC-Ag and pre-Cyfra levels were significant predictors of PFS. High $\frac{{\Delta \log \left( {{\text{SCC-Ag}}} \right)}}{{\Delta {\text{time}}}}$(post) and $\frac{{\Delta \log \left( {{\text{Cyfra}}} \right)}}{{\Delta {\text{time}}}}$(post) showed a tendency toward increased risk for PFS (*P* = 0.059; *P* = 0.054, respectively). Among group comparison of Kaplan–Meier plots revealed significant differences in DSS and PFS rates among the low-, intermediate-, and high-risk groups (Fig. 3). The 5DSS rates in the low-, intermediate-, and high-risk groups were 100%, 90.6%, and 62.5%, respectively (*P* < 0.0001). The 5PFS rates in the low-, intermediate-, and high-risk groups were 100%, 79.5%, and 50%, respectively (*P* = 0.022).

**Table 2:** Multivariate analysis for disease specific survival and progression free survival.

Factors	Disease specific survival		Progression free survival	
	Hazard ratio (95% CI)	*P*	Hazard ratio (95% CI)	*P*
Age (≥ 65 years)	2.5 (0.9–7.0)	0.079		
Treatment time (≥ 56 days)	2.1 (0.8–5.4)	0.136	1.8 (0.93–3.6)	0.08
NLR (≥ 2.35)	2.0 (0.78–5.1)	0.148	1.7 (0.84–3.3)	0.142
Pre-SCC-Ag (≥ 5 ng/ml)	10.1 (2.98–34.3)	< 0.001	3.7 (1.43–9.8)	0.007
Pre-Cyfra (≥ 3.15 ng/ml)	4.2 (1.17–15.2)	0.028	5.3 (2.09–13.3)	< 0.001
$\frac{{\Delta \log \left( {{\text{SCC-Ag}}} \right)}}{{\Delta {\text{time}}}}$(mid) (≥ − 5.524)			1.9 (0.89–3.9)	0.099
$\frac{{\Delta \log \left( {{\text{SCC-Ag}}} \right)}}{{\Delta {\text{time}}}}$(post) (≥ − 1.575)	6.0 (1.64–21.9)	0.007	2.7 (0.96–7.7)	0.059
$\frac{{\Delta \log \left( {{\text{Cyfra}}} \right)}}{{\Delta {\text{time}}}}$(post) (≥ − 0.697)	3.0 (0.82–11.3)	0.097	2.5 (0.98–6.3)	0.054

CI: confidence interval, SCC-Ag: squamous cell carcinoma antigen, Cyfra: carcinoembryonic antigen 21-1, pre-SCC-Ag: pretreatment SCC-Ag, pre-Cyfra: pretreatment Cyfra, $\frac{{\Delta \log \left( {{\text{SCC-Ag}}} \right)}}{{\Delta {\text{time}}}}$(mid): average log change rate × 100 (pre-SCC-Ag ~ mid-SCC-Ag), $\frac{{\Delta \log \left( {{\text{SCC-Ag}}} \right)}}{{\Delta {\text{time}}}}$(post): average log change rate × 100 (pre-SCC-Ag ~ post-SCC-Ag), $\frac{{\Delta \log \left( {{\text{Cyfra}}} \right)}}{{\Delta {\text{time}}}}$(post): average log change rate × 100 (pre-Cyfra ~ post-Cyfra).

## Discussion

This study investigated whether pre-SCC-Ag levels and the average log change rate after treatment could predict the treatment outcomes in patients with stage IIIC1 cervical SCC treated with CCRT. The results suggested that incorporating both the pre-SCC-Ag levels and the average rate of log change between pre- and post-SCC-Ag levels can lead to improved predictive accuracy for treatment outcomes in patients with stage IIIC1 cervical SCC. Pre-SCC-Ag levels and the average rate of log change in pre-SCC-Ag levels, based on an exponential decrease, were identified as potential biomarkers that can facilitate clinical decision-making in this heterogeneous group of patients.

Previous studies have predominantly focused on the prognostic utility of pre- and post-treatment SCC-Ag levels^8^. However, prognosis based on SCC-Ag levels during or after treatment includes the pre-SCC-Ag levels and the rate of change in SCC-Ag levels following treatment, which presents challenges in accurately predicting the mortality risk. Therefore, we proposed the use of pre-SCC-Ag levels and treatment-dependent change rates as better prognostic predictors in patients with stage IIIC1 SCC. The rate of change in SCC-Ag levels following anticancer radiotherapy is typically measured as the decrease rate at a specific time point $\left( {\frac{{\Delta {\text{SCC-Ag}}}}{{{\text{pre SCC}} - {\text{Ag}}}}} \right)$ compared with that before treatment^9^. Furthermore, post-treatment SCC-Ag measurements were conducted clinically at various time points (Fig. 1A), and post-CCRT tumor reduction tends to follow an exponential rather than a linear distribution^12,13^. Therefore, the rate of log change over time, expressed as $\frac{{\Delta \log \left( {{\text{SCC-Ag}}} \right)}}{{\Delta {\text{time}}}}$, is considered a variable that accurately reflects the real treatment response. The combination of pre-SCC-Ag levels and $\frac{{\Delta \log \left( {{\text{SCC-Ag}}} \right)}}{{\Delta {\text{time}}}}$(post) demonstrated superior performance as indicated by AUC and Youden index values on the ROC curves, outperforming combinations that included the reduction rate or linear change rate and showed a high mortality risk. Kaplan–Meier plots, stratified according to the combination of pre-SCC-Ag levels and the log change rate of post-treatment SCC-Ag levels, revealed significant differences in DSS and PFS. This indicated that this combination has a superior predictive power for assessing treatment outcomes. The log change rate of post-SCC-Ag levels provided an accurate reflection of treatment response, considering that the response to CCRT can continue even after treatment completion, as previously reported^13,14^. The time required to achieve complete remission after CCRT completion has been reported to be 3.5 months in patients with rectal cancer and is 3 months according to the kinetic model of tumor volume for cervical cancer^13,14^. These findings highlight the importance of considering posttreatment changes in SCC-Ag levels for accurate evaluation of treatment responses and prediction of patient outcomes.

Previous studies have reported that pre-Cyfra is a biomarker that complements pre-SCC-Ag in the prognostic prediction of cervical cancer^15,16^. Consistent with these findings, the combination of pre-SCC-Ag and pre-Cyfra showed a higher AUC and Youden index on the ROC curve than pre-SCC-Ag alone; moreover, it significantly predicted DSS in the multivariate analysis. However, pre-Cyfra and $\frac{{\Delta \log \left( {{\text{Cyfra}}} \right)}}{{\Delta {\text{time}}}}$(post) did not improve the AUC and could not significantly predict DSS in the multivariate analysis, suggesting that SCC-Ag better reflects post-treatment tumor burden reduction compared with Cyfra. The high degree of sensitivity of SCC-Ag to CCRT and the rapid change in SCC-Ag levels during treatment supported this hypothesis (Fig. 1C,D).

A previous study on the risk of stage IIIC1 cervical cancer in patients treated with CCRT reported that tumor size and the number of pelvic LN metastases were more significant predictors of disease-free survival than LE^17^. However, another study reported that patients with parametrial involvement (PMI) had a worse prognosis than those without PMI, suggesting a prognostic effect of PMI on LE^4^. These conflicting findings suggest that tumor size, number of LN metastases, and LE, as determined by imaging studies, may not consistently predict survival outcomes. Therefore, the current study, in which we focused on evaluating pre-SCC-Ag levels and the average rate of log change in post-SCC-Ag levels, presents an advanced method for assessing the pre-treatment disease extent and the treatment response. By incorporating these factors, we aimed to accurately stratify the mortality risk. This approach provided a comprehensive evaluation that extends beyond traditional imaging-based assessments. Pre- SCC-Ag levels and the dynamic changes in SCC-Ag levels may be reliable and consistent prognostic indicators of survival in patients with stage IIIC1 cervical SCC undergoing CCRT. Moreover, patients identified as high-risk may have a significantly increased risk (≈ 50%) of distant metastasis. In case they can conveniently visit the hospital, they can opt for systemic evaluations such as abdominal/chest CT or PET-CT every 1–3 months. This facilitates prompt initiation of further treatment upon recurrence. In cases where regular follow-up appointments pose challenges, additional chemotherapy may be considered as a viable option. Conversely, for patients classified as low-risk, annual follow-up appointments may suffice, with tests such as chest CT being considered optional. Patients categorized as intermediate-risk are monitored every 3–4 months, with abdominal/chest CT or PET-CT being deemed appropriate when TM levels are elevated or on an annual basis.

However, this study has several limitations. First, the lack of continuous and sufficient measurements of SCC-Ag levels during and after CCRT impedes confirmation of the exponential change and only allows for estimation. Second, the current method could not predict disease progression prior to post-SCC-Ag measurements. Third, the number of low- and high-risk groups categorized by pre-SCC-Ag levels, and the average rate of log change in post-SCC-Ag levels, was too small, which may limit the reliability of results. Finally, this study was a retrospective analysis based on data collected over a long period. Despite these limitations, this study presented a promising approach for evaluating treatment response using the log change rate after pre-SCC-Ag assessment and improving the predictive power for treatment outcomes in patients with stage IIIC1 cervical cancer.

In conclusion, during risk stratification analysis, incorporating both pre-SCC-Ag levels and the average rate of log change in SCC-Ag in the pre- and post-treatment stages is predicted to provide superior predictive ability for treatment outcomes of in patients with cervical SCC undergoing CCRT compared with using pre-SCC-Ag levels alone. However, further validation using large prospective studies is necessary to confirm these results and establish the clinical utility of this approach.

### Supplementary Information

Supplementary Information.

## Data Availability

Coding, supplement material, and dataset are available in https://github.com/oyeoncho/scc_ag.
