# Supplementary material for: Average log change rate of pretreatment squamous cell carcinoma antigen after concurrent chemoradiotherapy in stage IIIC1 cervical squamous cell carcinoma
Source: Sci Rep. 2024 Apr 15;14:8710. doi: 10.1038/s41598-024-59412-w (PMC11018847; doi:10.1038/s41598-024-59412-w)
Supplement: Supplementary file 1 — Supplementary Information. [file 41598_2024_59412_MOESM1_ESM.docx]

Supplementary Materials

| Table S1. Sensitivity, specificity, and Youden index of receiver operating characteristic (ROC) curves, as well as the *P*-value between ROC curves | | | | | |
| --- | --- | --- | --- | --- | --- |
| Factor1 | Factor2 | Sensitivity | specificity | Youden index | *P* value |
| Pre-SCC-Ag |  | 0.75 | 0.635 | 0.385 |  |
| Pre-SCC-Ag | $\frac{\Delta\log\left( SCC-Ag \right)}{\Delta time}$(mid) | 0.8 | 0.561 | 0.361 | 0.77 (vs. pre SCC-Ag) |
| Pre-SCC-Ag | $\frac{\Delta\log\left( SCC-Ag \right)}{\Delta time}$(post) | 0.75 | 0.716 | 0.466 | 0.436 (vs. pre SCC-Ag) |
| Pre-SCC-Ag | $\frac{\Delta SCC-Ag}{pre SCC-Ag}$ (mid) | 0.75 | 0.601 | 0.351 | 0.783 (vs. pre SCC-Ag) |
| Pre-SCC-Ag | $\frac{\Delta SCC-Ag}{pre SCC-Ag}$ (post) | 0.7 | 0.635 | 0.335 | 0.966 (vs. pre SCC-Ag) |
| Pre-SCC-Ag | $\frac{\Delta SCC-Ag}{\Delta time}$ (mid) | 0.75 | 0.608 | 0.358 | 0.875 (vs. pre SCC-Ag) |
| Pre-SCC-Ag | $\frac{\Delta SCC-Ag}{\Delta time}$ (post) | 0.6 | 0.777 | 0.377 | 0.751 (vs. pre SCC-Ag) |
| Pre-Cyfra |  | 0.85 | 0.432 | 0.282 |  |
| Pre-Cyfra | $\frac{\Delta\log\left( Cyfra \right)}{\Delta time}$(mid) | 0.7 | 0.568 | 0.268 | 0.658 (vs. pre Cyfra) |
| Pre-Cyfra | $\frac{\Delta\log\left( Cyfra \right)}{\Delta time}$(post) | 0.45 | 0.872 | 0.322 | 0.951 (vs. pre Cyfra) |
| Pre-Cyfra | $\frac{\Delta Cyfra}{\mathrm{pre}Cyfra}$ (mid) | 0.65 | 0.628 | 0.278 | 0.77 (vs. pre Cyfra) |
| Pre-Cyfra | $\frac{\Delta Cyfra}{\mathrm{pre}Cyfra}$ (post) | 0.75 | 0.486 | 0.236 | 0.217 (vs. pre Cyfra) |
| Pre-Cyfra | $\frac{\Delta Cyfra}{\Delta time}$(mid) | 0.35 | 0.865 | 0.215 | 0.312 (vs. pre Cyfra) |
| Pre-Cyfra | $\frac{\Delta Cyfra}{\Delta time}$(post) | 0.85 | 0.432 | 0.282 | 0.352 (vs. pre Cyfra) |
| Pre-SCC-Ag | Pre-Cyfra | 0.55 | 0.845 | 0.395 | 0.568 (vs. pre SCC-Ag) |
| Pre-SCC-Ag: pretreatment squamous cell carcinoma antigen, pre-Cyfra: pretreatment carcinoembryonic antigen 21-1, $\frac{\Delta\log\left( SCC-Ag \right)}{\Delta time}$(mid): average log change rate x 100 (pre-SCC-Ag ~ mid-SCC-Ag), $\frac{\Delta\log\left( SCC-Ag \right)}{\Delta time}$ (post): average log change rate x 100 (pre-SCC-Ag ~ post-SCC-Ag), $\frac{\Delta\log\left( \mathrm{Cyfra} \right)}{\Delta time}$(mid): average log change rate x 100 (pre-Cyfra ~ mid-Cyfra), $\frac{\Delta\log\left( \mathrm{Cyfra} \right)}{\Delta time}$(post): average log change rate x 100 (pre-Cyfra ~ post-Cyfra) | | | | | |

| Table S2. univariate analysis for disease specific survival and progression free survival | | | | |
| --- | --- | --- | --- | --- |
|  | Disease specific survival | | Progression free survival | |
| Factors | Hazard ratio (95% CI) | *P* | Hazard ratio (95% CI) | *P* |
| Age ($\geq$65 years) | 2.75 (1.21~6.75) | 0.028 | 1.67 (0.82~3.4) | 0.16 |
| EQD2 (< 70 Gy) | 1.34 (0.55~3.25) | 0.523 | 0.54 (0.26~1.14) | 0.105 |
| Treatment time ($\geq$56 days) | 1.64 (0.68~3.94) | 0.269 | 1.77 (0.93~3.37) | 0.084 |
| Local extension (Parametrium) | 0.85 (0.24~2.96) | 0.797 | 1.85 (0.56~6.11) | 0.314 |
| Local extension (PW/LV) | 0.97 (0.19~4.79) | 0.966 | 3.81 (1.01~14.38) | 0.048 |
| Hemoglobin (< 11.75 g/dl) | 1.69 (0.68~4.25) | 0.262 | 1.63 (0.84~3.18) | 0.148 |
| NLR ($\geq$2.35) | 1.84 (0.73~4.62) | 0.194 | 1.85 (0.94~3.64) | 0.074 |
| Platelet ($\geq$269.5 x10^3^/µL) | 0.52 (0.21-1.31) | 0.169 | 0.91 (0.48-1.74) | 0.782 |
| Pre-SCC-Ag ($\geq5 ng/ml)$ | 2.88 (1.05~7.93) | 0.041 | 1.87 (0.95~3.69) | 0.069 |
| Mid-SCC-Ag ($\geq1.25$ng/ml) | 2.27 (0.87~5.92) | 0.092 | 1.99 (1.01~3.91) | 0.046 |
| Post-SCC-Ag ($\geq0.8 ng$/ml) | 1.69 (0.69~4.15) | 0.254 | 1.23 (0.64~2.35) | 0.535 |
| Pre-Cyfra ($\geq3.15 ng/ml)$ | 2.47 (0.95~6.43) | 0.064 | 2.63 (1.3~5.34) | 0.007 |
| Mid-Cyfra ($\geq1.5 ng/ml)$ | 1.68 (0.67~4.2) | 0.271 | 1.67 (0.85~3.28) | 0.138 |
| Post-Cyfra ($\geq1.2 ng/ml)$ | 1.84 (0.73~4.63) | 0.192 | 1.7 (0.87~3.35) | 0.123 |
| $\frac{\Delta\log\left( SCC-Ag \right)}{\Delta time}$(mid) ($\geq$-5.524) | 0.87 (0.36~2.1) | 0.757 | 1.25 (0.66~2.39) | 0.496 |
| $\frac{\Delta\log\left( SCC-Ag \right)}{\Delta time}$(post) ($\geq$-1.575) | 1.34 (0.55~3.22) | 0.521 | 0.99 (0.52~1.89) | 0.974 |
| $\frac{\Delta\log\left( \mathrm{Cyfra} \right)}{\Delta time}$(mid) ($\geq$-2.976) | 0.52 (0.21~1.3) | 0.16 | 0.75 (0.39~1.44) | 0.388 |
| $\frac{\Delta\log\left( \mathrm{Cyfra} \right)}{\Delta time}$(post) ($\geq$-0.697) | 1.58 (0.65~3.87) | 0.315 | 0.99 (0.52~1.89) | 0.977 |
| CI: confidence interval, SCC-Ag: squamous cell carcinoma antigen, Cyfra: carcinoembryonic antigen 21-1, pre-SCC-Ag: pretreatment SCC-Ag, mid-SCC-Ag: SCC-Ag during treatment, post-SCC-Ag: posttreatment SCC-Ag, pre-Cyfra: pretreatment Cyfra, mid-Cyfra: Cyfra during treatment, post-Cyfra: posttreatment Cyfra, $\frac{\Delta\log\left( SCC-Ag \right)}{\Delta time}$(mid): average log change rate x 100 (pre-SCC-Ag ~ mid-SCC-Ag), $\frac{\Delta\log\left( SCC-Ag \right)}{\Delta time}$(post): average log change rate x 100 (pre-SCC-Ag ~ post-SCC-Ag), $\frac{\Delta\log\left( \mathrm{Cyfra} \right)}{\Delta time}$(mid): average log change rate x 100 (pre-Cyfra ~ mid-Cyfra), $\frac{\Delta\log\left( \mathrm{Cyfra} \right)}{\Delta time}$(post): average log change rate x 100 (pre-Cyfra ~ post-Cyfra) | | | | |

Fig. S1. Receiver operating characteristic curves and corresponding AUCs with 95% confidence intervals. (A) Pre-SCC-Ag and reduction rates during and after treatment. (B) Pre-Cyfra and reduction rates during and after treatment. (C) Pre-SCC-Ag and average linear change rates during and after treatment. (D) Pre-Cyfra average linear change rates during and after treatment.


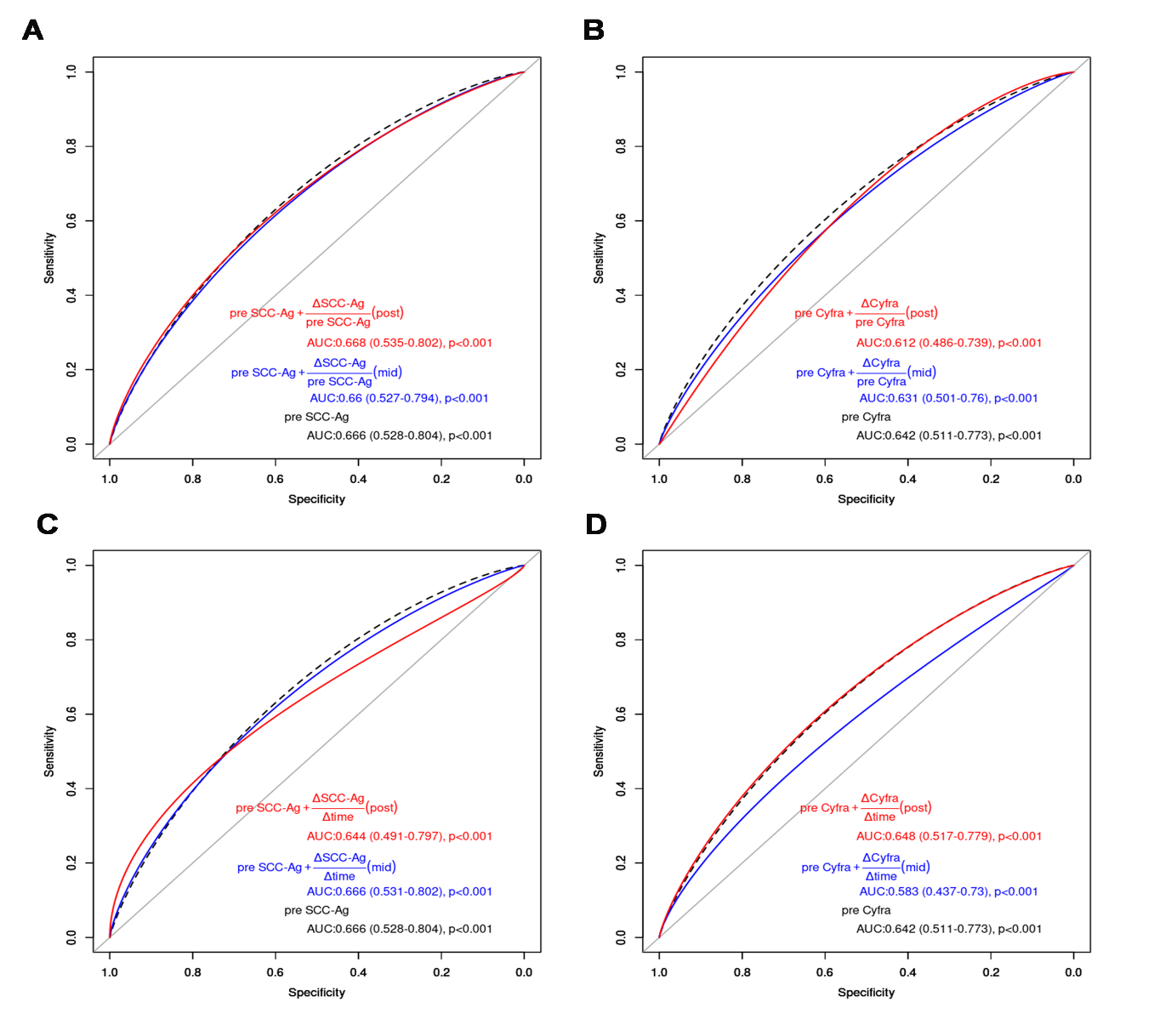


AUC: area under the curve; pre SCC-Ag: pretreatment squamous cell carcinoma antigen; pre Cyfra: pretreatment carcinoembryonic antigen 21-1.
